# Fucoxanthin inhibits cardiac fibroblast transdifferentiation by alleviating oxidative stress through downregulation of BRD4

**DOI:** 10.1371/journal.pone.0291469

**Published:** 2023-09-12

**Authors:** Jinxia Han, Yanfang Zhang, Haisheng Peng

**Affiliations:** 1 Shaoxing Seventh People’s Hospital, Shaoxing, China; 2 Department of pharmacology, Medical college, Shaoxing University, Shaoxing, China; University of Rochester Medical Center, UNITED STATES

## Abstract

Myocardial fibrosis can lead to ischemic damage of the myocardium, which can be life-threatening in severe cases. Cardiac fibroblast (CF) transdifferentiation is an important process in myocardial fibrosis. Fucoxanthin (FX) plays a key role in ameliorating myocardial fibrosis; however, its mechanism of action is not fully understood. This study investigated the role of FX in the angiotensin II (Ang II)-induced transdifferentiation of CFs and its potential mechanisms of action. We found that FX inhibited Ang II-induced transdifferentiation of CFs. Simultaneously, FX downregulated bromodomain-containing protein 4 (BRD4) expression in CFs and increased nuclear expression of nuclear factorerythroid 2-related factor 2 (Nrf2). FX reverses AngII-induced inhibition of the Keap1/Nrf2/HO-1 pathway and elevates the level of reactive oxygen species (ROS). FX failed to reverse Ang II-induced changes in fibrosis-associated proteins and ROS levels after Nrf2 silencing. BRD4 silencing reversed the inhibitory effect of Ang II on the Keap1/Nrf2/HO-1 antioxidant signalling pathway. In conclusion, we demonstrated that FX inhibited Ang II-induced transdifferentiation of CFs and that this effect may be related to the activation of the Keap1/Nrf2/HO-1 pathway by reducing BRD4 expression and, ultimately, oxidative stress.

## Introduction

Cardiovascular diseases (CVDs) are known as the "first killers, endangering human health [1]. Myocardial fibrosis is a common result of various CVDs, including hypertension, cardiac insufficiency, arrhythmia, and ischemic cardiomyopathy [2–4]. Cardiac fibrosis is a cardiac-interstitial remodelling characterized by the excessive proliferation of cardiac fibroblasts (CFs), excessive deposition, and abnormal distribution of collagen [5]. Under pathological conditions, CFs transdifferentiate into myofibroblasts, which highly express α-smooth muscle actin (α-SMA) and oversecrete collagens I and II, fibronectin (FN), and other extracellular matrices (ECMs) [6, 7]. These changes in the ECM lead to an imbalance in collagen synthesis and degradation, eventually leading to myocardial fibrosis [6, 7]. Myocardial fibrosis can lead to cardiac dysfunction and metabolic abnormalities [8]. Therefore, the prevention and treatment of myocardial fibrosis are of great significance for improving CVDs.

Oxidative stress is an important inducer of fibrosis in multiple tissues, including the myocardium [9, 10]. Oxidative stress produces a large amount of reactive oxygen species (ROS), which induces CF proliferation and ECM remodelling [9, 10]. Keap1/Nrf2 is an important signalling pathway involved in intracellular anti-oxidative stress [11, 12]. Under normal physiological conditions, Keap1 binds Nrf2 and inhibits its activity. After stimulation by oxidative stressors, Keap1 is uncoupled from Nrf2, and Nrf2 enters the nucleus and regulates the transcription of downstream genes, thereby exerting antioxidant effects [13, 14]. Ghrelin inhibits the NADPH/ROS pathway by activating Nrf2, ultimately improving myocardial fibrosis [15]. Keap1/Nrf2 may participate in myocardial fibrosis by inhibiting oxidative stress. BRD4 plays an important role as a transcriptional regulator in cancers, and autoimmune and inflammatory diseases. BRD4 inhibition downregulates Keap1 expression and activates Nrf2, thereby attenuating hydrogen peroxide-induced oxidative stress injury in trophoblast cells [16]. Inhibition of BRD4 attenuates TGF-β-induced endothelial-mesenchymal transition and cardiac fibrosis [17] and BRD4 may act as a Keap1/Nrf2 upstream regulatory target to regulate oxidative stress and myocardial fibrosis.

Fucoxanthin (FX) is a carotenoid derived from *Sargassum horneri*, *Dictyota coriacea*, and the brown seaweed wakame (*Undaria pinnatifida*) [18]. Fucoxanthin exerts antibacterial, anti-inflammatory, antidepressant, antitumor, and neuroprotective effects [18], reduces oxidative stress and alleviates renal fibrosis by regulating Nrf2 expression [19]. It can also inhibit renal fibrosis by alleviating oxidative stress through the Akt/Sirt1/FoxO3α signalling pathway [20]. Studies on pulmonary fibrosis have demonstrated that FX inhibits α-SMA, collagen I, and fibronectin expression *in vitro* and attenuates bleomycin-induced pulmonary fibrosis *in vivo* [21]. These findings suggest that FX plays an important role in alleviating renal and pulmonary fibrosis. However, their role in myocardial fibrosis remains unclear. In this study, we investigated the effects of FX on angiotensin II-induced cardiac fibroblast transdifferentiation and explored the potential role and signalling mechanisms of the BRD4/Keap1/Nrf2 pathway.

## Materials and methods

### Isolation, purification, and culture of primary CFs

Neonatal Sprague-Dawley rats aged 1–2 days were euthanized by dislocation, and heart tissue was excised, followed by digestion with 0.08% trypsin (HyClone, Logan, UT, USA) and 0.06% type II collagenase (Solarbio, China). The digested cells were sieved through a 150-μm mesh to remove large pieces of tissue and subsequently cultured in DMEM (Sigma-Aldrich, St. Louis, MO, USA) containing 10% fetal bovine serum (FBS, Gibco) in a 5% CO_2_ incubator (Thermo Fisher, Waltham, MA, USA) at 37°C for 1 h. The supernatant was discarded to remove nonadherent endothelial cells, vascular smooth muscle cells, pericytes, and dead cells. The medium was replaced with DMEM containing 10% FBS to continue the culture. CFs were cultured until the second generation for follow-up studies. All experiments involving animals were approved by the hospital ethics committee and met ethical requirements (approval number:2021-0507-01; approval date:2021-05-07).

### Identification of CFs by immunofluorescence

Cardiac fibroblast were identified using immunofluorescence. The CFs were inoculated into cell-imaging dishes and incubated overnight. Cardiac fibroblasts were then treated with 4% paraformaldehyde (Beyotime, China), 0.2% Triton X-100 (Beyotime), and 5% bovine serum albumin (BSA) (Beyotime) for 20, 5, and 30 min, respectively. Cardiac fibroblast were then incubated with primary antibodies against desmin (1:50), alkaline phosphatase (1:50), von Willebrand factor (vWF) (1:200), vimentin (1:100), Nrf2 (1:100), and BRD4 (1:50) (Absin, China) overnight at 4°C. The cells were then incubated with goat anti-mouse IgG/PE secondary antibody (1:250) (Absin), goat anti-rabbit IgG/Alexa Fluor 647 secondary antibody, or goat anti-mouse IgG/Alexa Fluor 488 secondary antibody (1:500) (Invitrogen, Carlsbad, CA, USA) for 1 h. Fluorescence quencher (containing 5 mg/mL DAPI) (Solarbio) was added to the samples before visualization. Cells were imaged using an IX71 inverted fluorescence microscope (Olympus, Tokyo, Japan).

### MTT analysis

The cells were treated with Ang II (1–10000 nM; MedChem Express, Monmouth Junction, NJ, USA) and/or FX (0–160 μM; Lot number: HY-N2302-27529; purity: 99.17%; MedChem Express), for 18~48 h and then 10 μL MTT (5 mg/mL; Solarbio) was added and the samples were incubated for 4 h. Crystals were dissolved in dimethyl sulfoxide (Solarbio). A Synergy H1 Microplate Detector (BioTek, Winooski, VT, USA) was used to measure absorbance of the 96-well plates.

### Wound healing experiment

Cells were seeded in 12-well plates and cultured overnight. When the cells reached 100% confluence, scratching was performed, floating cells were washed away, and the wound/scratch area was treated with Ang II and/or FX. Scratches were photographed using an inverted microscope (Olympus) at 0 h and at the end of the experiment.

### Western blot

Total and nuclear proteins were extracted using RIPA lysis buffer and a nucleoprotein and Cytoplasmic Protein Extraction Kit (Beyotime), respectively, according to the manufacturer’s instructions. Proteins were quantified and separated by sodium dodecyl sulfate-polyacrylamide gel (SDS-PAGE) electrophoresis. Primary antibodies were as follows: α-SMA (1:1000), collagen I (1:1000), collagen II (1:1000), FN (1:1000), Nrf2 (1:1000), Keap1 (1:1000), GAPDH (1:2000),-tubulin (1:2000), and LaminB1 (1:1000) (Cell Signalling Technology, Danvers, MA, USA). The secondary antibodies used were goat anti-rabbit IgG-HRP and goat anti-mouse IgG-HRP (Absin). After washing-off the secondary antibodies, an enhanced chemiluminescence kit (Thermo Fisher Scientific) was used to detect primary and secondary antibody complexes. Gels were visualized using a GelDoc XR+ gel layer imaging system (Bio-Rad, Hercules, CA, USA) and gray-scale values of the proteins were analyzed using ImageJ 1.8.0.

### Hydroxyproline colorimetric assay

Cells were treated with Ang II and/or FX, and the cell culture supernatants were separated. The colorimetric assay was performed using a hydroxyproline test kit (Solarbio), according to the manufacturer’s instructions.

### Flow cytometry

Cells were collected in 5 mL centrifuge tubes, washed twice with PBS, fixed in 70% ethanol at 4°C for 30 min, and centrifuged at 1500×*g* for 5 min. Ethanol was washed-off with PBS, centrifuged at 1500×*g* for 5 min, PBS was discarded, and 300 μL PI/RNase staining working solution (Solarbio) was added and incubated in the dark for 60 min at 22°C. Detection was performed using flow cytometry (BD Biosciences, San Jose, CA, USA) with an excitation of 488 nm.

### Detection of ROS by DCFH-DA fluorescent probe method

Cells were treated with Ang II and/or FX. The DCFH-DA (Solarbio) was diluted according to the manufacturer’s instructions and used for ROS detection. Cell fluorescence was visualized using an IX71 inverted fluorescence microscope (Olympus).

### Transfection with small interfering RNA (siRNA) to silence Nrf2 or BRD4 protein expression

Cells were grown to 50% confluence and transfected using an Nrf2 or BRD4 siRNA transfection kit (Genomeditech, China). The negative control (NC) and Nrf2 or BRD4 siRNAs (150 pM) were diluted in 75 μL DMEM. Entranster-R4000 (3 μL) was diluted with 72 μL of DMEM. The dilutions were mixed and allowed to stand for 15 minutes. Diluent mixtures were added to the cells and incubated for 24 h. Western blotting was performed to detect Nrf2 or BRD4 protein silencing. Nrf2 siRNA sequences: sense, 5’-GCAGCAAACAAGAGAUGGCAATT-3’; anti-sense, 5’-UUGCCAUCUCUUGUUUGCUGCTT-3. BRD4 siRNA sequences: sense, 5’- GCGUUUCCACGGUACCAAATT-3, ‘ anti-sense, 5’-UUUGGUACCGUGGAAACGCTT-3’.

### Statistical analyses

All data are expressed as mean ± standard deviation. Comparisons among multiple groups were performed using one-way analysis of variance (ANOVA), followed by Dunnett’s test using GraphPad Prism 9.0 (GraphPad Software, CA, USA). Statistical significance was set at *P* < 0.05.

## Results

### Ang II induces transdifferentiation of CFs

**Fig 1:**
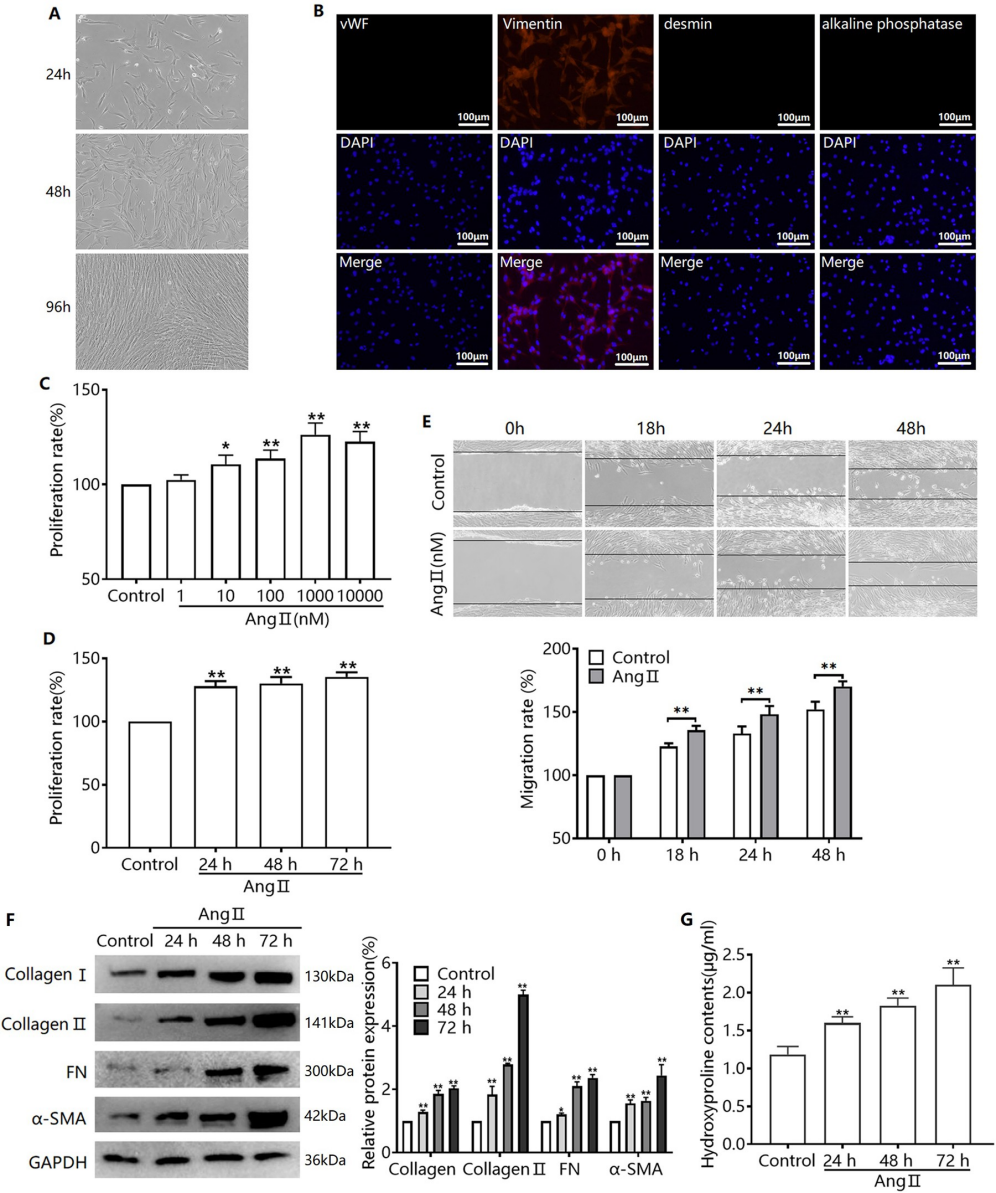
Ang II induces the conversion of CFs to MF. (A) Cell morphology of CFs grown for 24, 48, and 96 h. (B) The expression of vWF, vimentin, desmin, and alkaline phosphatase proteins in primary CFs was detected by immunofluorescence. (C) CFs were treated with 1–10,000 nM Ang II and changes in cell proliferation capacity were detected by MTT assay. CFs were treated with 1000 nM Ang II for 24, 48, and 72 h, and changes in cell proliferation (D) and migration (E) capacities (E) were detected by MTT and wound healing assay. (F) Western blots were performed to detect the protein expression of collagens I and II, FN and α-SMA in each group. (G) The content of hydroxyproline in the supernatant of each group was measured with a hydroxyproline kit.^*^P < 0.05, ^**^P < 0.01 *versus* Control or 0 h.

Morphological observations revealed that primary CFs were mainly fusiform. The CFs were completely confluent at 72–96 h (Fig 1A). Isolated CFs may contain endothelial cells, muscle cells, and pericytes. The respective molecular markers used were vWF, desmin, and alkaline phosphatase. The higher the vimentin content, the lower the presence of vWF, desmin, and alkaline phosphatase, and the higher the purity of the CFs. Our study demonstrated that isolated CFs had a vimentin positivity rate above 95% and a total positivity rate for vWF, desmin, and alkaline phosphatase below 5% (Fig 1B). In this study, we successfully isolated CFs with high purity.

The conversion of CFs to myofibroblasts (MF) was induced by treating CFs with Ang II. We found that a specific dose of Ang II enhanced the proliferation and migration of CFs (Fig 1C–1E). Generally, the expression of α-SMA protein in cells is enhanced after the successful transformation of CFs into MF, and the levels of some ECMs, including collagens I and II, and FN, are elevated. Consequently, we examined the expression of these four proteins and demonstrated that Ang II enhanced the protein expression of α-SMA, collagens I, and II, and FN in CFs (Fig 1F). Simultaneously, the levels of hydroxyproline, a marker of collagen secretion, increased (Fig 1G). These results suggest that Ang II successfully induced the transformation of CFs into MF.

### FX inhibits Ang Ⅱ-induced transdifferentiation of CFs

**Fig 2:**
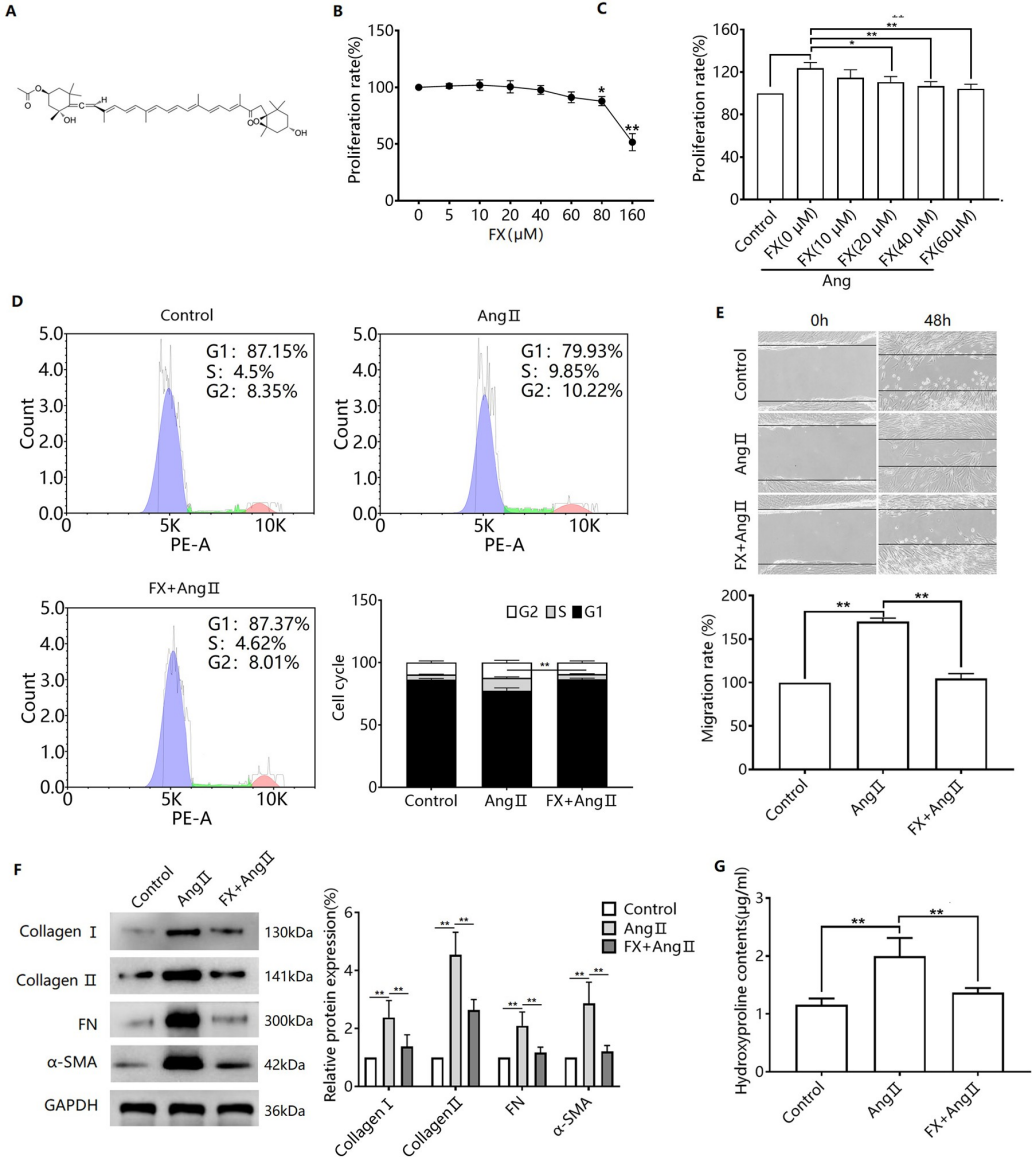
Effect of FX on Ang II-induced transdifferentiation of CFs. (A) FX chemical structure. (B) CFs were treated with 0–160 μM FX for 24 h, and cell proliferation was detected by MTT assay. ^*^P<0.05, ^**^P<0.01 *versus* FX (0 μM) (C) CFs were pretreated with FX (10, 20, and 40 μM) for 24 h. Cells were then treated with Ang II for 48 h and changes of cell proliferation were detected by MTT. ^*^P<0.05, ^**^P<0.01. Flow cytometry was used to detect cell cycle changes (D), and a wound healing assay was used to detect cell migration (E). Western blots were used to detect cellular collagens I and II, FN, α-SMA protein expression (F), and a hydroxyproline kit was used to detect hydroxyproline in the supernatant of CFs (G). ^**^P<0.01.

To determine the role of FX in Ang II-induced transformation of CFs, we investigated the effect of FX on CF proliferation. The chemical structure of FX is shown in Fig 2A. FX (0–40 μM) treatment for 24 h had no significant effect on the proliferation of CFs, while 80–160 μM FX inhibited the proliferation of CFs (Fig 2B). To exclude the effect of FX on cell proliferation, 40 μM FX was used in further experiments. Pretreatment with FX inhibited Ang II-induced increases in the proportion of S-phase cells in CFs, as well as their proliferative and migratory activities (Fig 2C–2E). FX pretreatment also inhibited Ang II-induced upregulation of collagens I and II, FN, and α-SMA protein expression and increased hydroxyproline content (Fig 2F and 2G).

### FX reverses Ang II-induced oxidative stress via the Keap1/Nrf2 pathway

**Fig 3:**
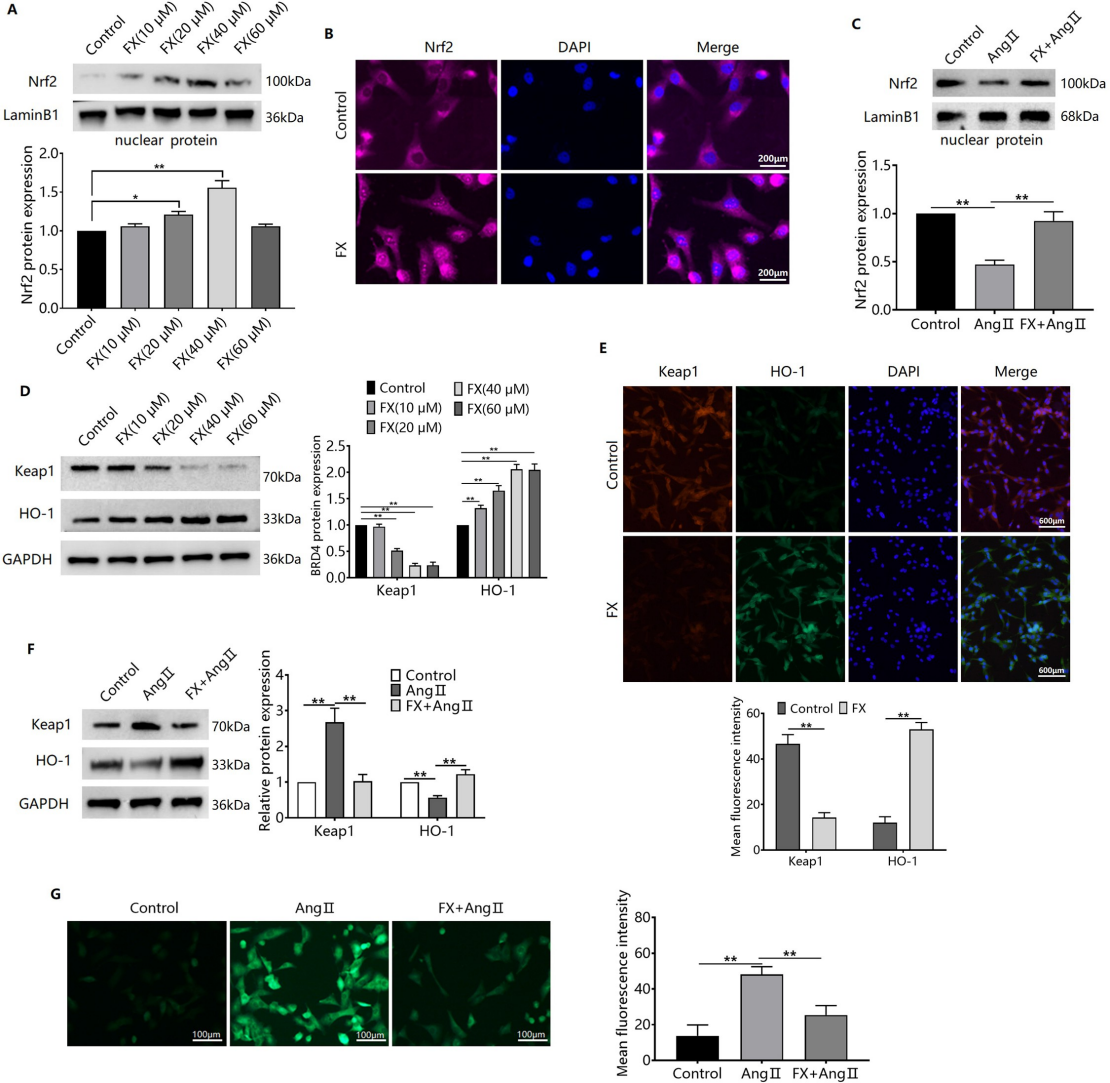
Effect of FX reversal of Ang II on the Keap1/Nrf2 pathway. (A) CFs were treated with FX (10, 20, 40, 60 μM) for 24 h and Western blots were used to detect Nrf2 protein expression in the cell nucleus of the CFs. (B) CFs were treated with FX (40 μM) for 24 h and immunofluorescence was used to detect Nrf2 protein expression in the cell nucleus of CFs. (C) Western blots were used to detect nuclear Nrf2 protein expression in Ang II- or/and FX-treated CFs. (D) CFs were treated with FX (10, 20, 40, 60 μM) for 24 h and Western blots were used to detect Keap1 and HO-1 protein expression. (E) CFs were treated with FX (40 μM) for 24 h and immunofluorescence was used to detect Keap1 and HO-1 protein expression. Western blots were used to detect nuclear Nrf2 protein expression in Ang II- and FX-treated CFs. Total cellular protein (F), and ROS level levels (G) of Keap1 and HO-1.^**^P<0.01.

Fucoxanthin upregulated Nrf2 protein expression in the nuclei of CFs (Fig 3A and 3B) and reversed the Ang II-induced downregulation of nucleolar Nrf2 protein expression in CFs (Fig 3C). Fucoxanthin also upregulated HO-1 protein expression and downregulated Keap1 protein expression in CFs (Fig 3D and 3E) and, reversed Ang II-induced upregulation of Keap expression and downregulation of HO-1 expression in CFs (Fig 3F). Since the Keap1/Nrf2/HO-1 signalling pathway has antioxidative stress effects, we examined the ROS levels and demonstrated that FX inhibited Ang II-induced elevation of ROS levels in CFs (Fig 3G).

### Silencing of Nrf2 inhibits FX resistance to Ang II-induced fibrosis-associated protein expression and elevated ROS in CFs

**Fig 4:**
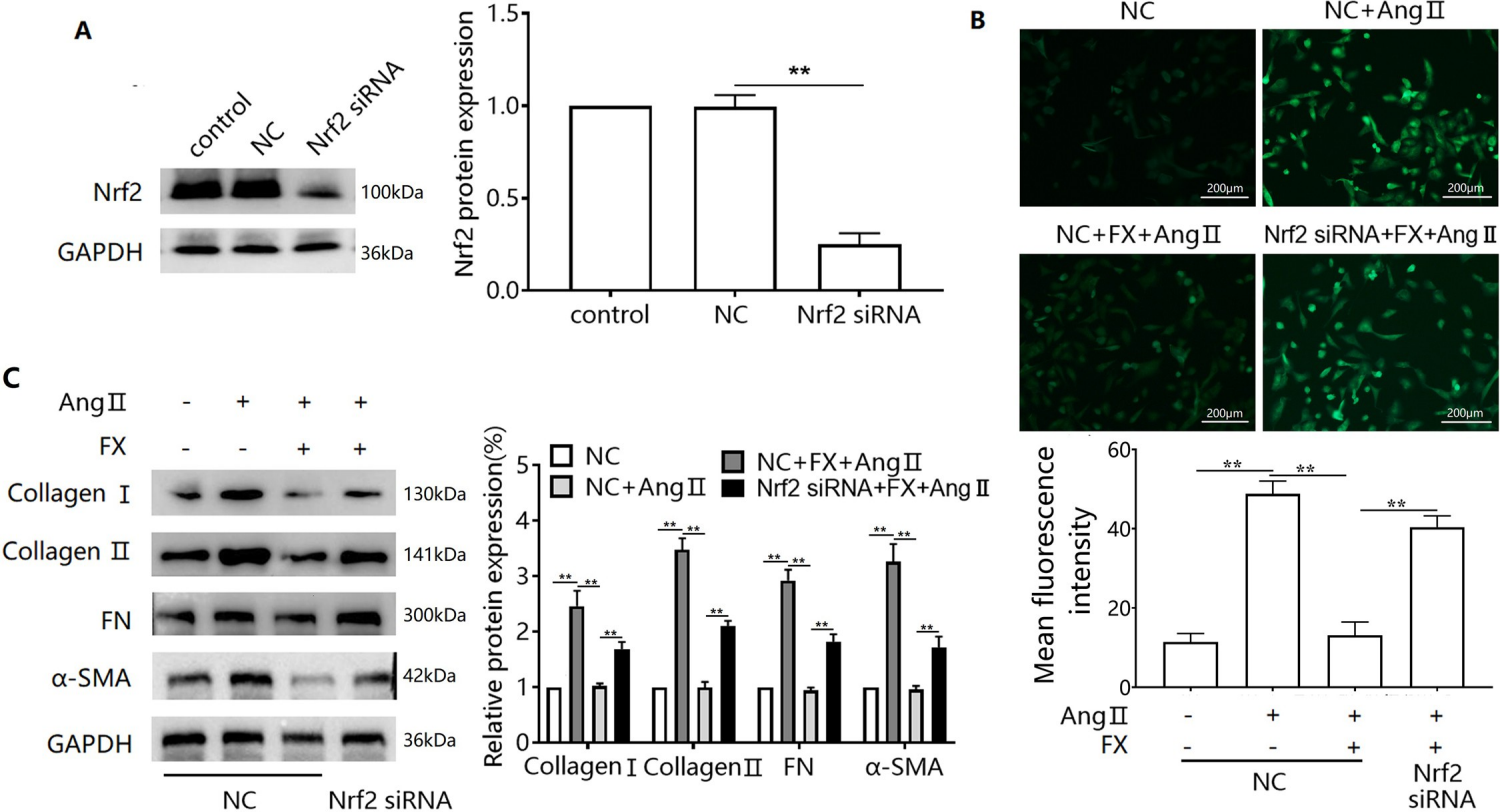
Effect of Nrf2 silencing on FX resistance to Ang II-induced elevation of CFs fibrosis-associated protein expression and ROS. (A) siRNA was used to interfere with Nrf2 and Western blots were used to detect cellular Nrf2 protein expression. CFs were silenced with Nrf2 protein expression, pretreated for 24 h with FX (40 μM), and then treated with Ang II for 48 h. ROS levels were detected by DCFH-DA fluorescent probe assay (B), Collagens I, and II, FN, and α-SMA protein expression were detected by Western blotting (C). ^**^P<0.01.

To further examine the mediating role of the Keap1/Nrf2 pathway, we used siRNA to interfere with Nrf2 protein expression (Fig 4A) and observed changes in the expression of ROS and fibrosis-associated proteins. Transfection with Nrf2 siRNA inhibited FX expression by reversing the Ang II-induced ROS elevation (Fig 4B). Furthermore, silencing of the Nrf2 protein reduced the inhibitory effect of FX on Ang II-induced upregulation of collagens I, and II, FN, and α-SMA protein expression (Fig 4C).

### FX reverses the inhibitory effect of Ang II on the Keap1/Nrf2 pathway through downregulation of BRD4

**Fig 5:**
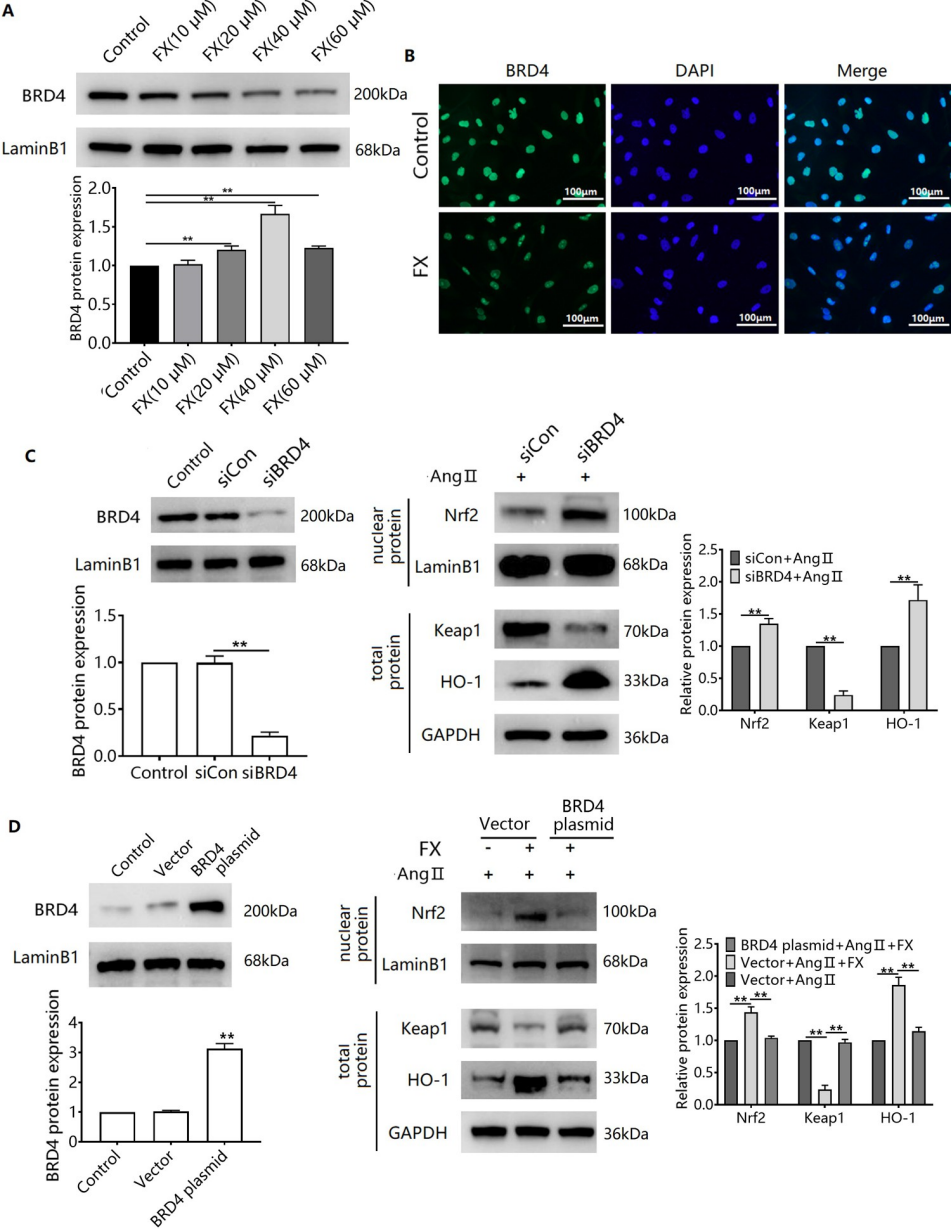
FX reverses the inhibitory effect of Ang II on the Keap1/Nrf2 pathway via BRD4. (A) CFs were treated with FX (5, 10, 20, 40 μM) for 24 h and Western blots were used to detect BRD4 protein expression. (B) CFs were treated with FX (40 μM) for 24 h and immunofluorescence was used to detect BRD4 protein expression. Silencing (C) and overexpression (D) of Nrf2 protein were followed by treatment with Ang II for 48 hours. Western blots were used to detect nuclear Nrf2 protein expression, and total cellular protein of Keap1 and HO-1.^**^P<0.01.

Fucoxanthin downregulated BRD4 protein expression in CFs (Fig 5A and 5B) and RNA interference of BRD4 expression reversed the Ang II-induced downregulation of nuclear Nrf2 protein expression in CFs (Fig 5C). Furthermore, silencing of BRD4 reversed Ang II-induced upregulation of Keap1 expression and downregulation of HO-1 expression in CFs (Fig 5C). To further verify the role of BRD4, we examined the effect of FX on Ang II induction by overexpressing BRD4. Overexpression of BRD4 reversed Ang II-induced Nrf2 expression in the nucleus of CFs, Ang II-induced downregulation of Keap1 protein expression, and upregulation of HO-1 protein expression (Fig 5D).

## Discussion

Ang II is an important active product of the renin-angiotensin-aldosterone system. It stimulates the release of epinephrine and norepinephrine, constricts blood vessels, and increases blood pressure [22, 23]. Ang II promotes synthesis and inhibits degradation of ECM, leading to accumulation of ECM and ultimately inducing renal [24], pulmonary [25], and myocardial fibrosis [26]. Cardiac fibrosis is a major contributor to heart disease and is accompanied by excessive activation of CFs by the generation of ECM [27]. In addition to cardiac fibrosis, Ang II induces cardiomyocyte hypertrophy and inflammation, leading to cardiac remodelling and knockdown of the Family with sequence similarity 114 member A1 (FAM114A1) gene which attenuates the induction of Ang II [28]. Therefore, effective prevention and treatment of Ang II-induced fibrosis is important.

Transdifferentiation of CFs into MFs is an important pathological process in myocardial fibrosis [29]. During the development of myocardial fibrosis, CFs proliferate and migration is increased [29]. Molecular changes in myocardial fibrosis are characterized by high expression of α-SMA, accompanied by increased secretion of ECM proteins, including collagens I and II, and FN [6, 7]. Additionally, hydroxyproline, a by-product of collagen breakdown, is expressed at elevated levels during the transdifferentiation of CFs [30]. Our study showed that Ang II induced an increase in the proliferation and migration of CFs, the proportion of S-phase cells, and the expression of α-SMA, collagens I and II, FN protein, and hydroxyproline. These findings suggest that Ang II induces transdifferentiation of CFs, whereas FX effectively inhibits the induction effect of Ang II.

The Keap1/Nrf2 antioxidant signalling pathway plays an important role in resistance to fibrosis. Various studies have shown that microRNA-26b inhibits isoproterenol-induced myocardial fibrosis and that this effect is mediated by the Keap1/Nrf2 signalling pathway [31], Keap1/Nrf2 plays a role in ameliorating DOX-induced myocardial fibrosis caused by H2S [32], and oxymatrine alleviates aldosterone-induced transdifferentiation of CFs into MFs *in vitro* by activating the Keap1/Nrf2 pathway [33]. These findings suggest that the Keap1/Nrf2 pathway plays an active role in resistance to myocardial fibrosis.

Fucoxanthin target Keap1 protein the Keap1/Nrf2 pathway to resist 6-hydroxydopamine-induced neuronal cell damage [34], and Nrf2 is a regulatory target of FX that mediates its anti-osteoclastogenic [35], anti-inflammatory [36], and anti-apoptotic [37] effects. Further, a study in diabetic rats showed that FX improves renal function and fibrosis by reducing oxidative stress through Nrf2 activation mediated by the silent information regulator T1 [19]. These findings suggest that FX inhibits Ang II-induced myocardial fibrosis via the Keap1/Nrf2 pathway. Our study found that FX upregulated Nrf2 protein expression and reversed Ang II-induced downregulation of Nrf2 expression in the nucleus of CFs. Fucoxanthin also reversed Ang II-induced upregulation of Keap1 and downregulation of HO-1. As an antioxidant effector, HO-1 reduces cellular ROS and positively regulates oxidative stress [38]. Our investigations of ROS showed that FX alleviated the Ang II-induced elevation of ROS in CFs. In addition, silencing of Nrf2 followed by FX treatment failed to reverse the Ang II-induced elevated expression of ROS and fibrosis markers. These findings suggest that FX reduces Ang II-induced oxidative stress by restoring the activity of the Keap1/Nrf2 pathway, thereby inhibiting the transdifferentiation of CFs.

We also observed that FX downregulated BRD4 protein expression in CFs. The inhibition of BRD4 reduces ROS production in prostate cancer cells, and this effect is associated with the Keap1/Nrf2 signalling pathway [39]. Furthermore, inhibition of BRD4 enhances Nrf2 activation through the downregulation of Keap1 expression, thereby attenuating hydrogen peroxide-induced oxidative stress damage in trophoblast cells [16]. Inhibition of BRD4 also attenuates TGF-β-induced endothelial-mesenchymal transition and cardiac fibrosis [17]. These findings suggest that FX exerts antioxidative stress effects by activating the Keap1/Nrf2 pathway through the inhibition of BRD4 expression. To further examine the role of BRD4 in FX inhibition of CFs transdifferentiation, we interfered with BRD4 expression using siRNA to observe the effect of Ang II on the Keap1/Nrf2 pathway in CFs. We showed that interference with BRD4 expression reversed Ang II-induced downregulation of nucleolar Nrf2 expression, and also reversed Ang II-induced upregulation of Keap1, and downregulation of HO-1, in CFs. These results suggest that FX reverses inhibition of the Keap1/Nrf2 pathway by Ang II, thereby reducing oxidative stress, which is associated with the downregulation of BRD4 expression. FX improves STZ-induced myocardial fibrosis in diabetic rats by attenuating oxidative stress and restoring mitosis [40].

The present study showed that FX alleviated oxidative stress and cardiac fibroblast transdifferentiation associated with BRD4. These results and associated research findings show FX to have potential in the pharmacological treatment of myocardial fibrosis.

## Conclusion

**Fig 6:**
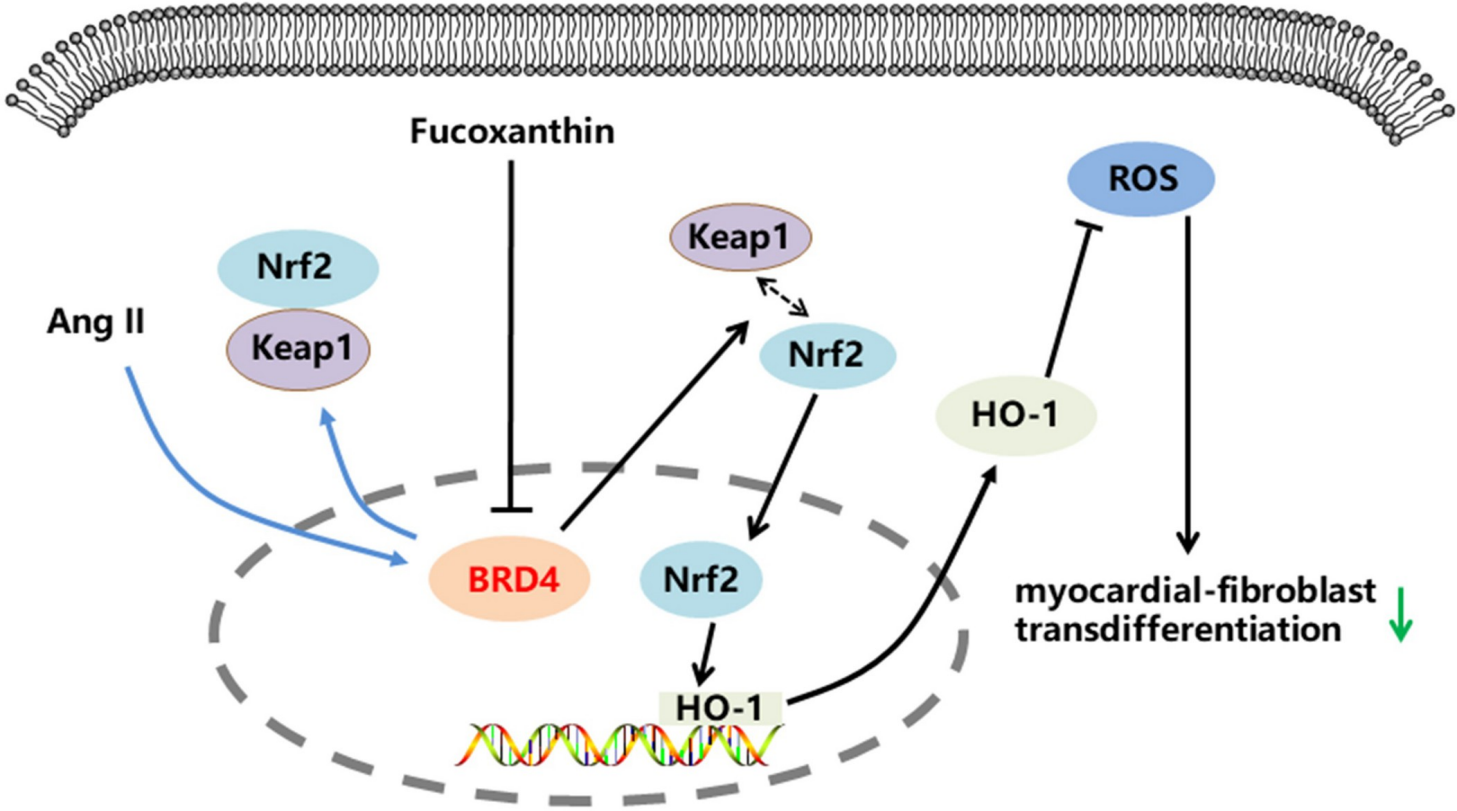
Signalling pathway of FX resistance to cardiac fibroblast transdifferentiation.

Our study showed that FX inhibits Ang II-induced transdifferentiation of CFs, and that this effect is associated with downregulation of BRD4 expression and thus activation of the Keap1/Nrf2 pathway, leading to a reduction in oxidative stress (Fig 6). Our study revealed a potential mechanism by which FX inhibits the transdifferentiation of CFs. These findings provide theoretical support for the treatment of myocardial fibrosis.

## Supporting information

S1 Raw images (PDF)
