# Supplementary material for: Fucoxanthin inhibits cardiac fibroblast transdifferentiation by alleviating oxidative stress through downregulation of BRD4
Source: PLoS One. 2023 Sep 12;18(9):e0291469. doi: 10.1371/journal.pone.0291469 (PMC10497131; doi:10.1371/journal.pone.0291469)
Supplement: S1 Raw images — (PDF) [file pone.0291469.s001.pdf]

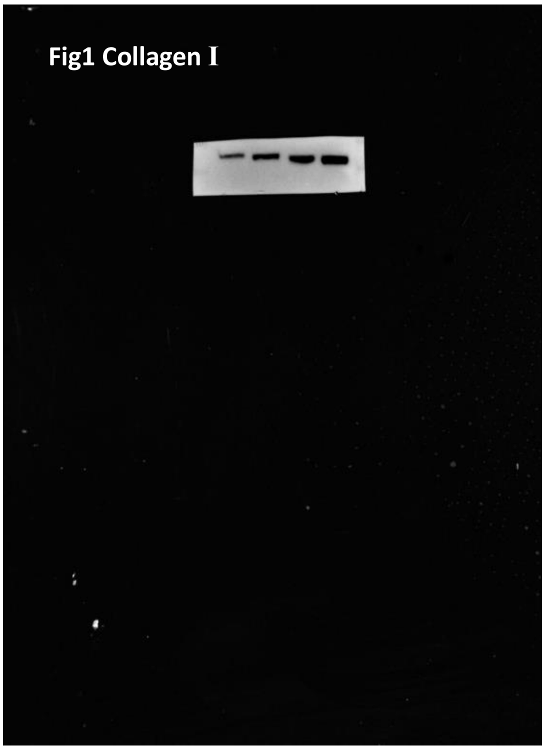
Fig1 Collagen I

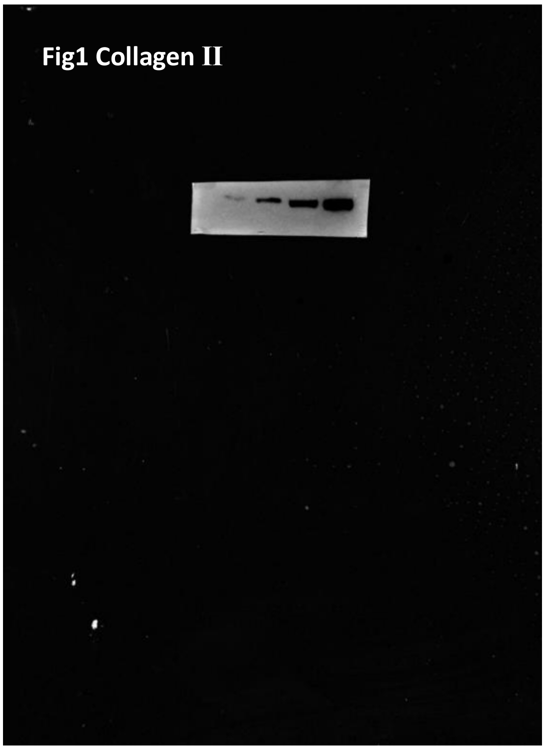
Fig1 Collagen II

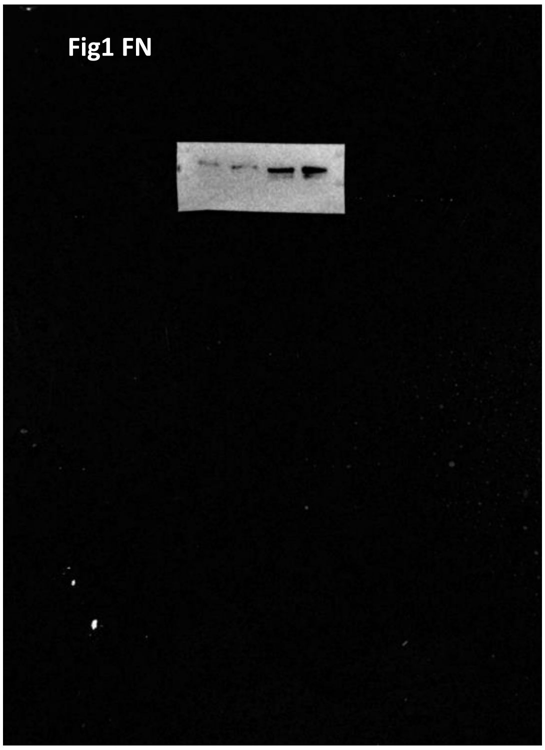
Fig1 FN

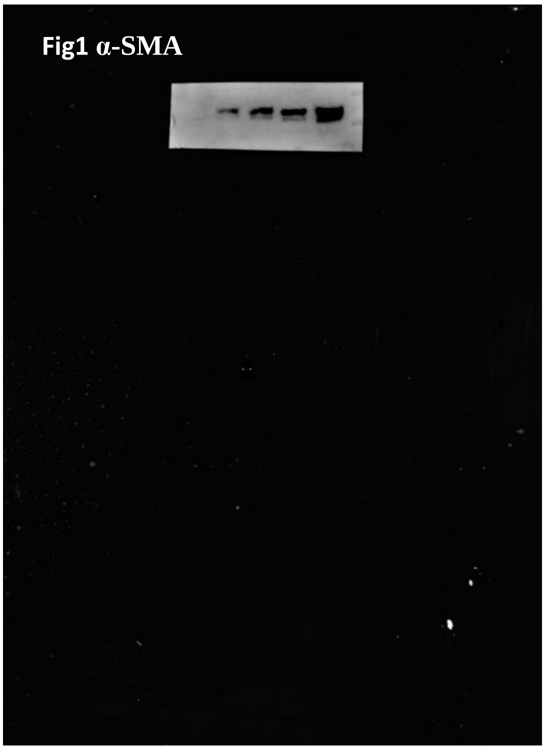
Fig1 α-SMA

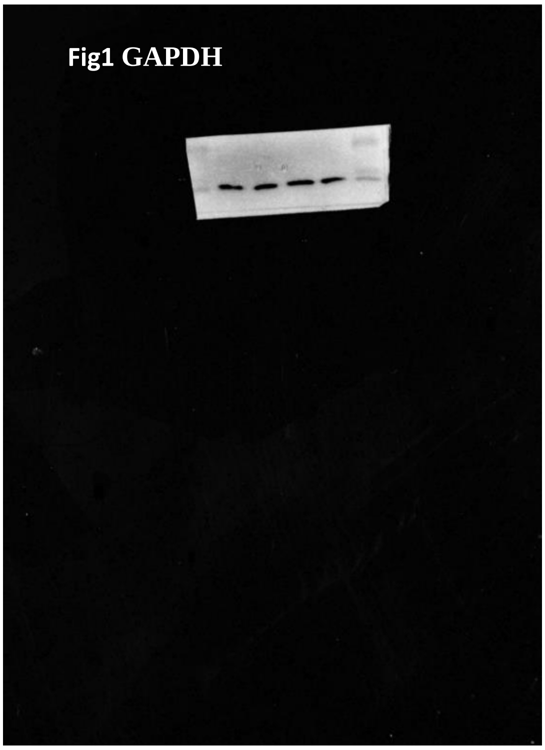
Fig1 GAPDH

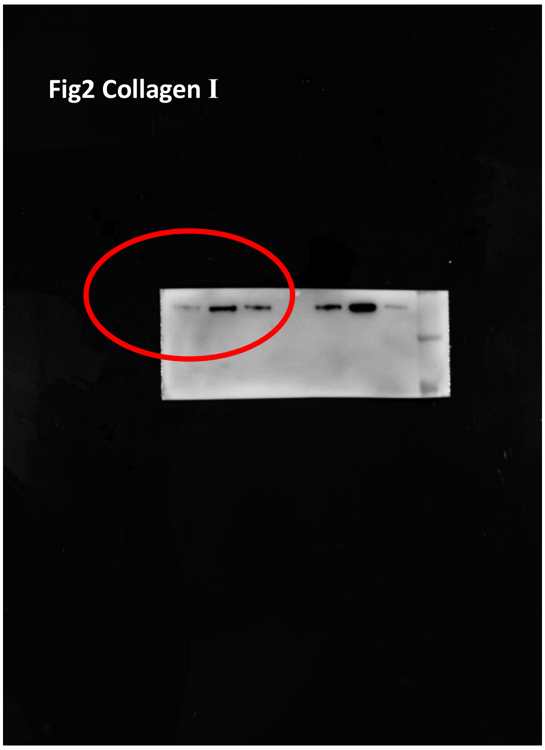
Fig2 Collagen I

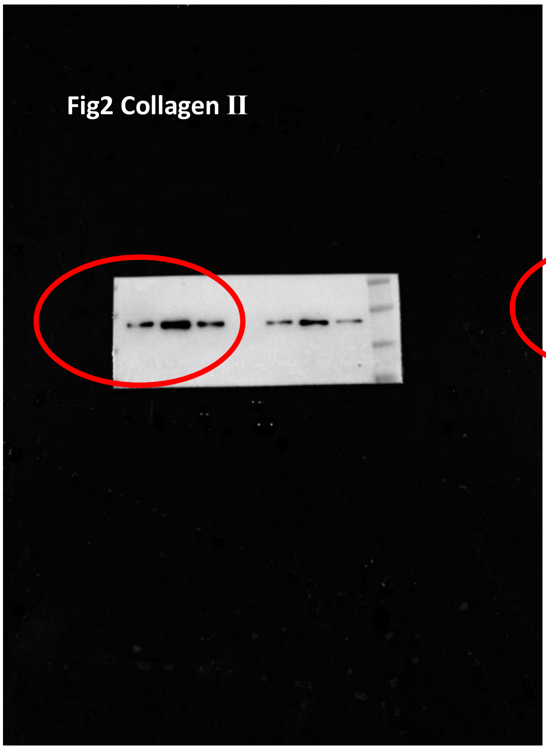
Fig2 Collagen II

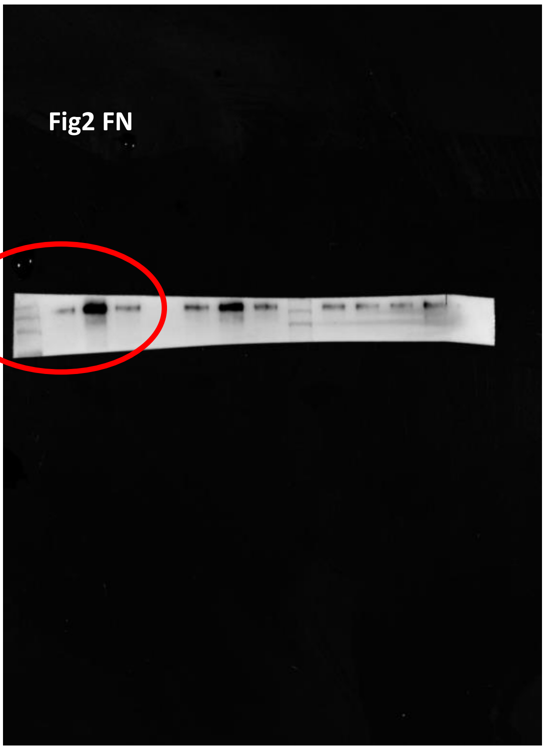
Fig2 FN

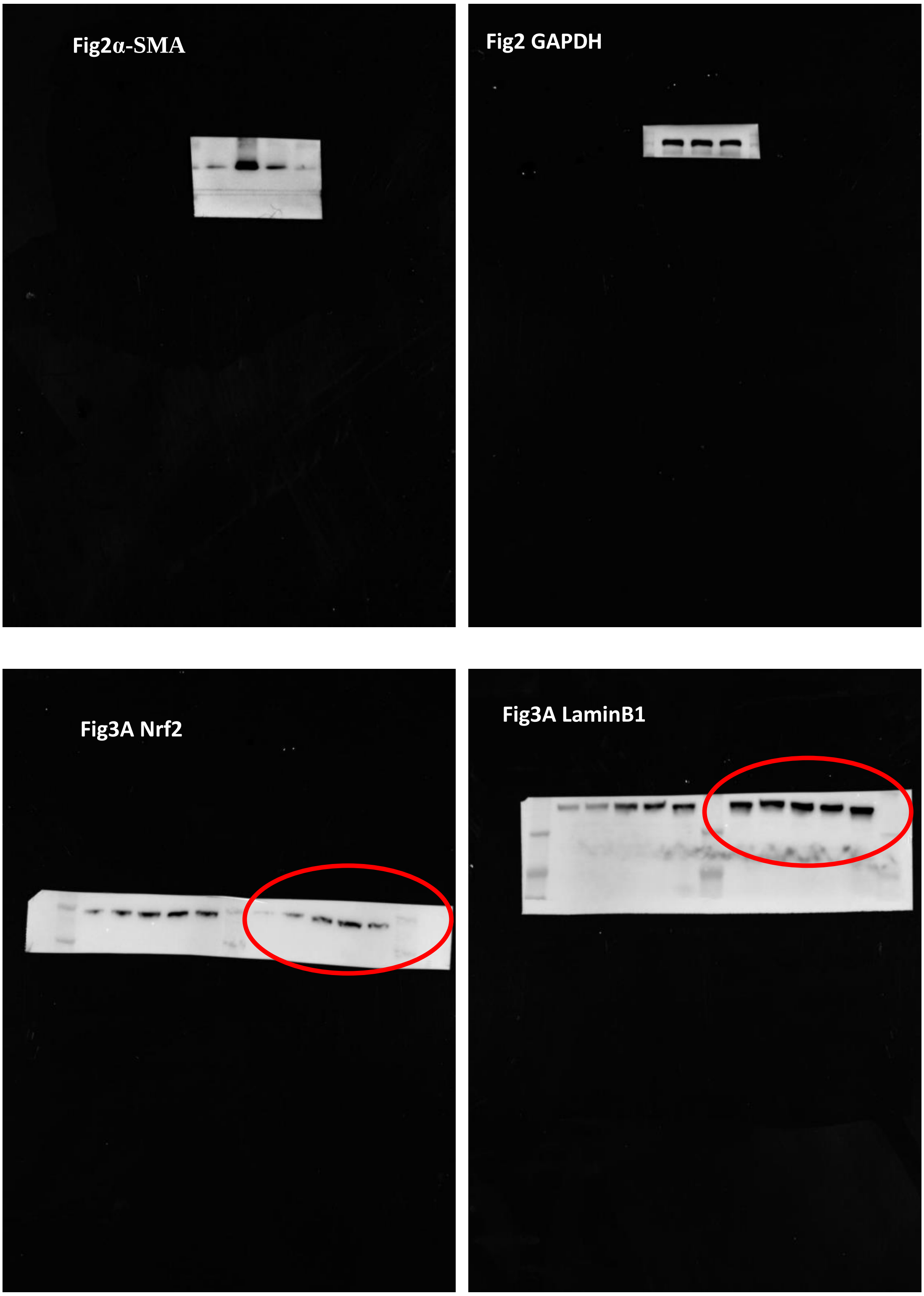

Figure3A

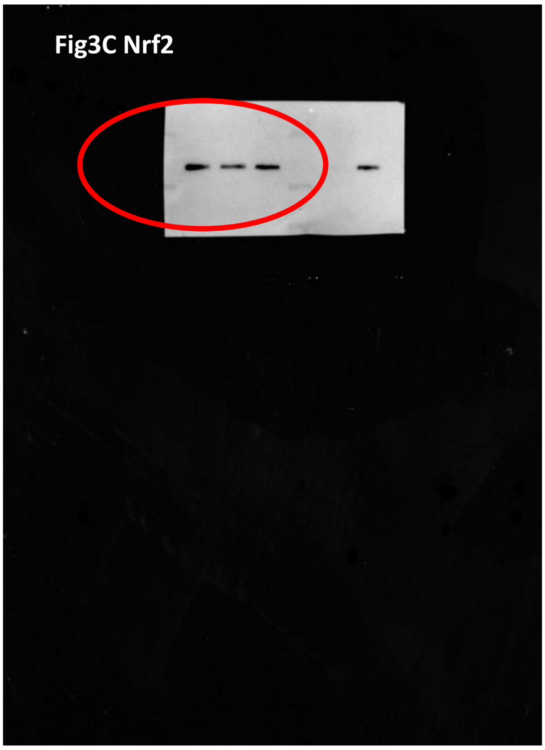
Fig3C Nrf2

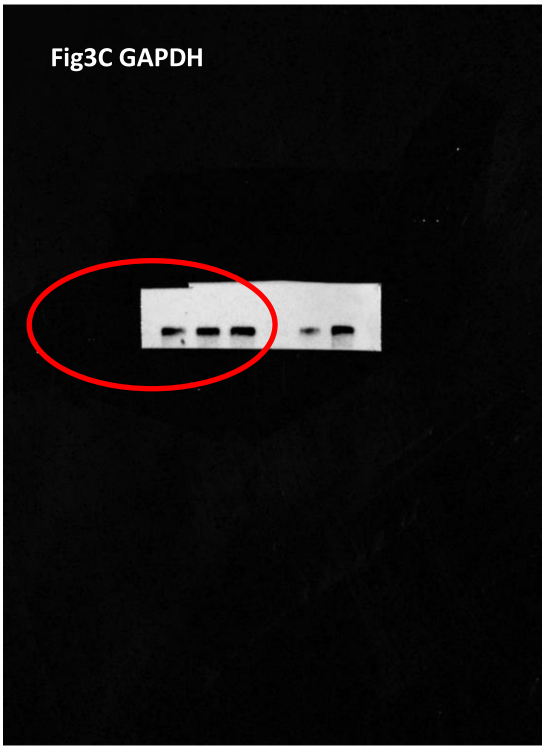
Fig3C GAPDH

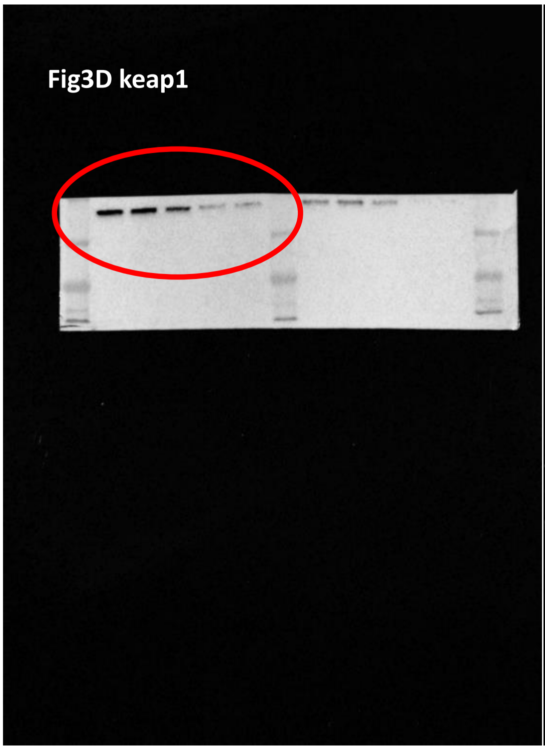
Fig3D keap1

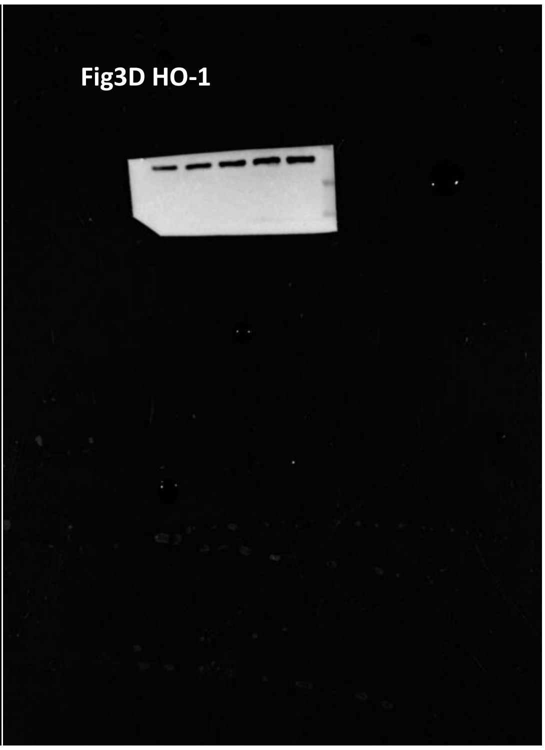
Fig3D HO-1

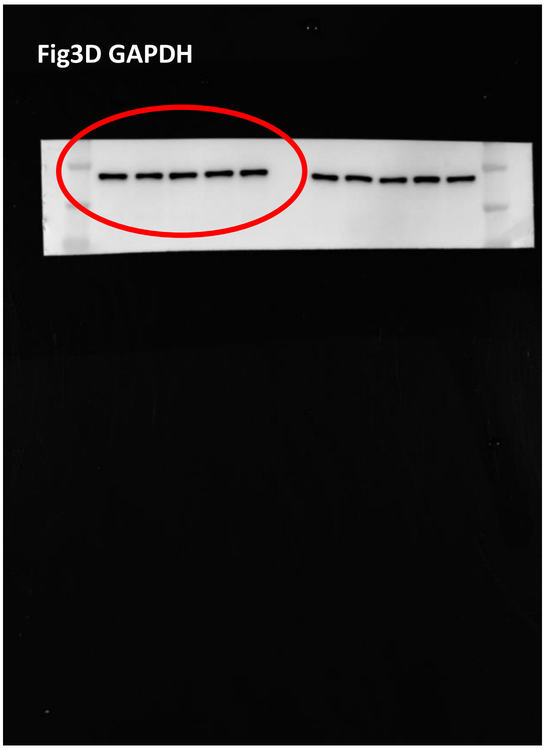
Fig3D GAPDH

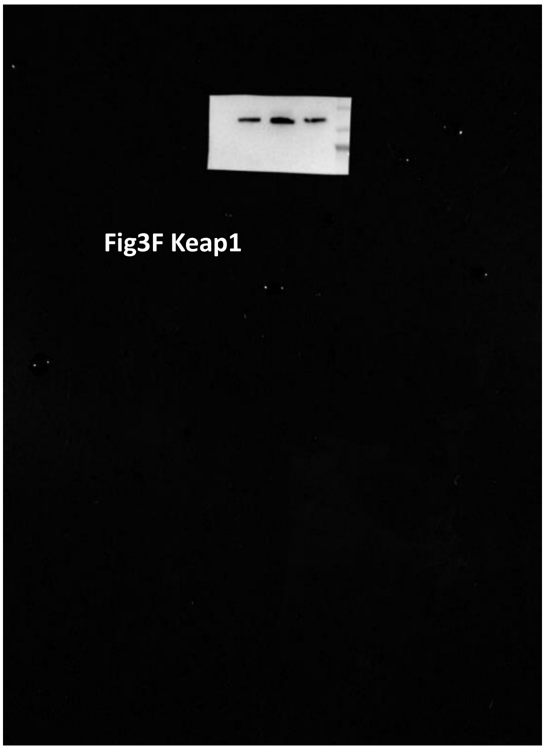
Fig3F Keap1

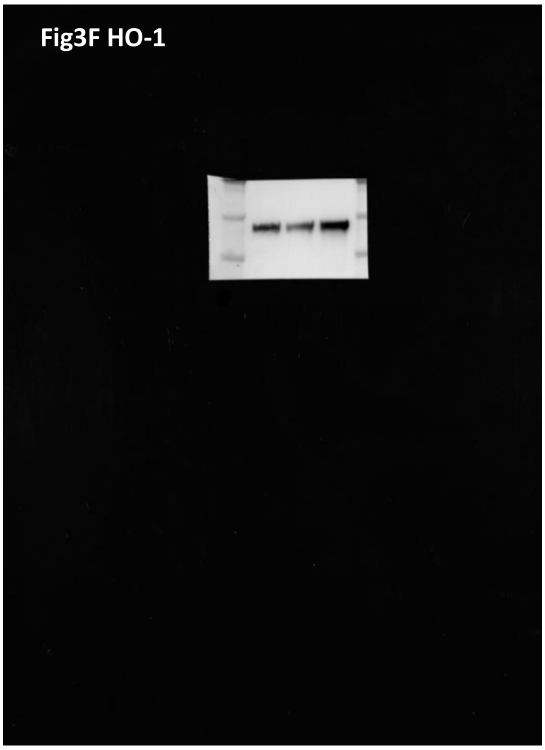
Fig3F HO-1

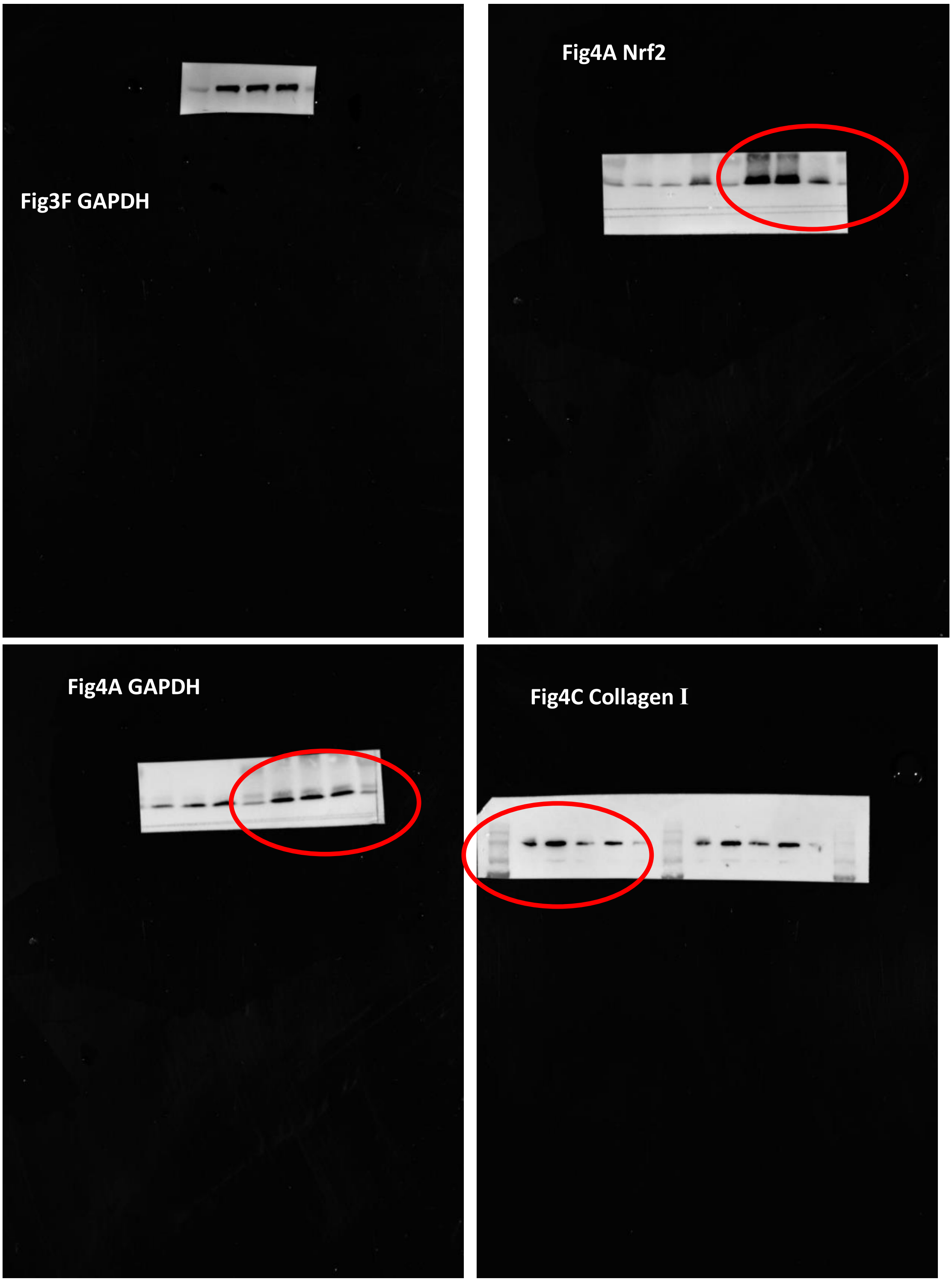
Fig3F GAPDH
Fig4A Nrf2
Fig4A GAPDH
Fig4C Collagen I

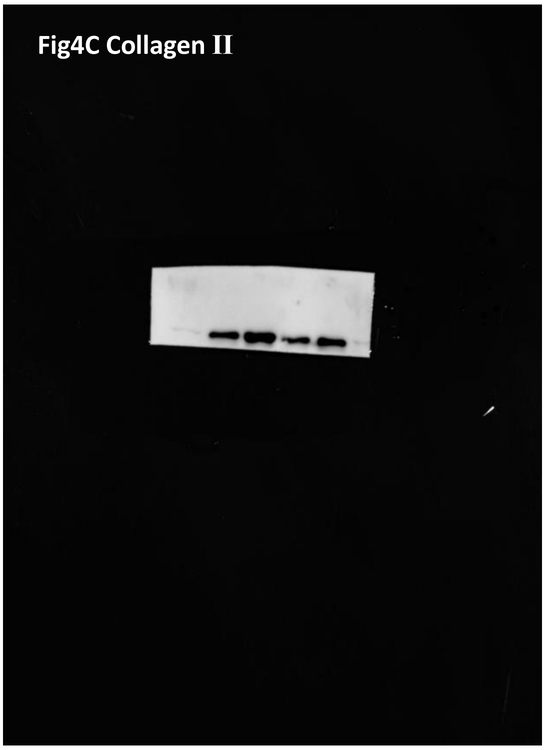
Fig4C Collagen II

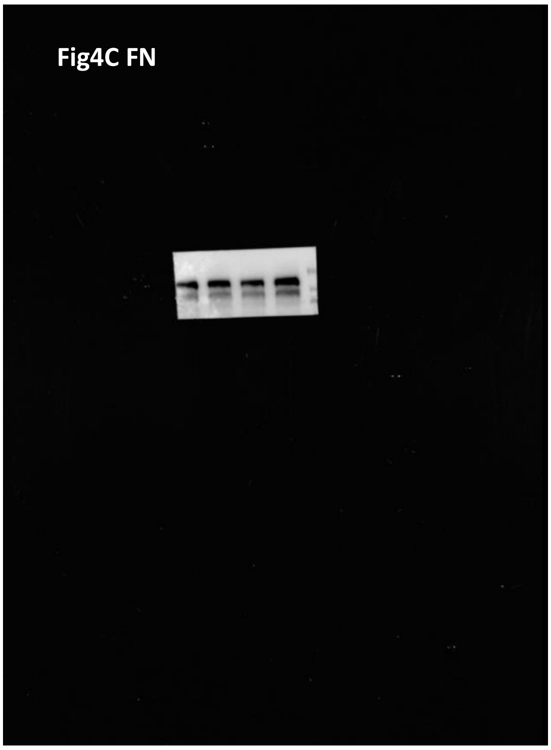
Fig4C FN

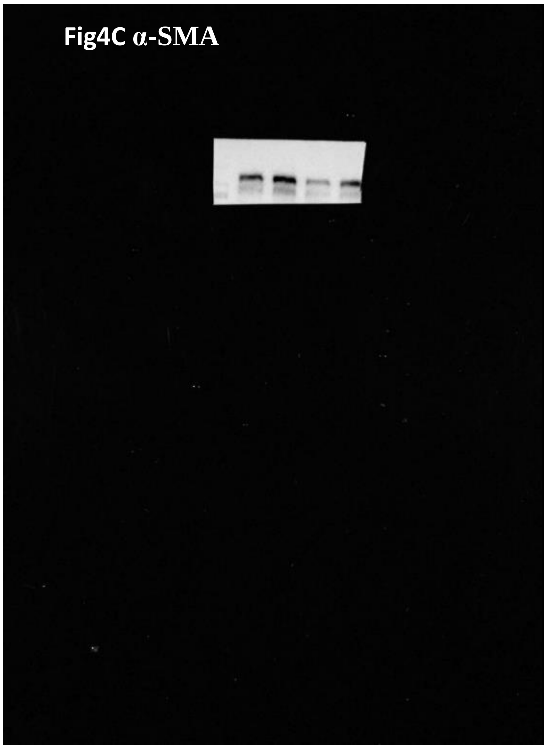
Fig4C α-SMA

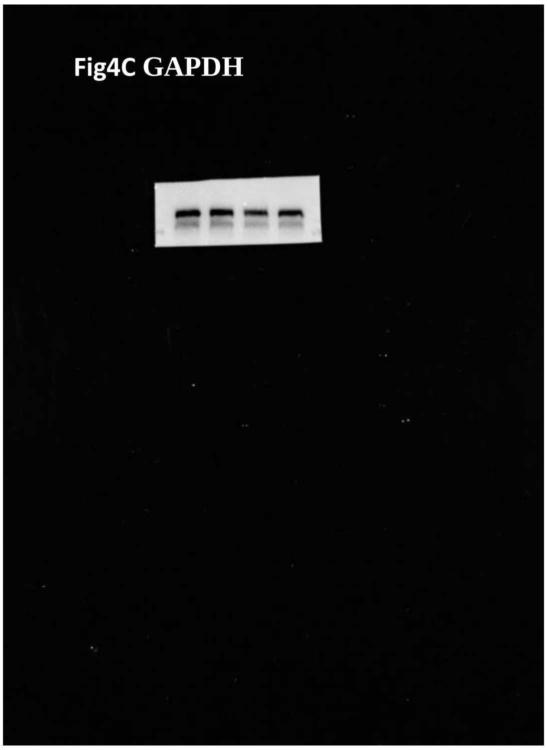
Fig4C GAPDH

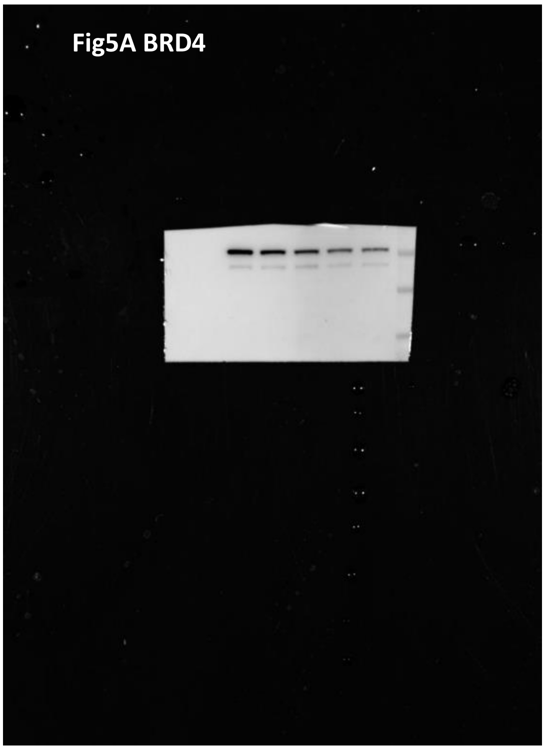
Fig5A BRD4

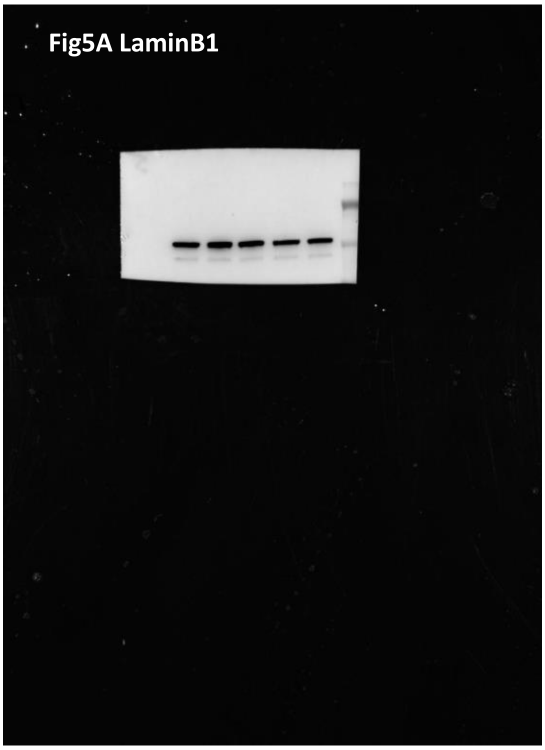
Fig5A LaminB1

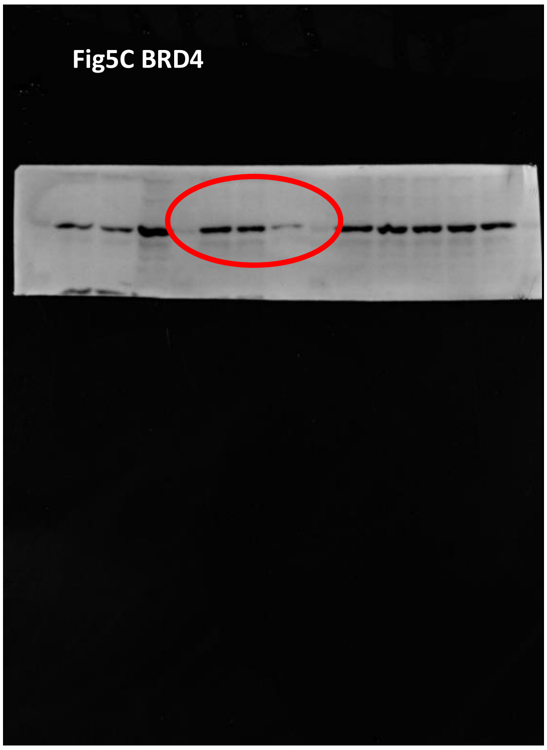
Fig5C BRD4

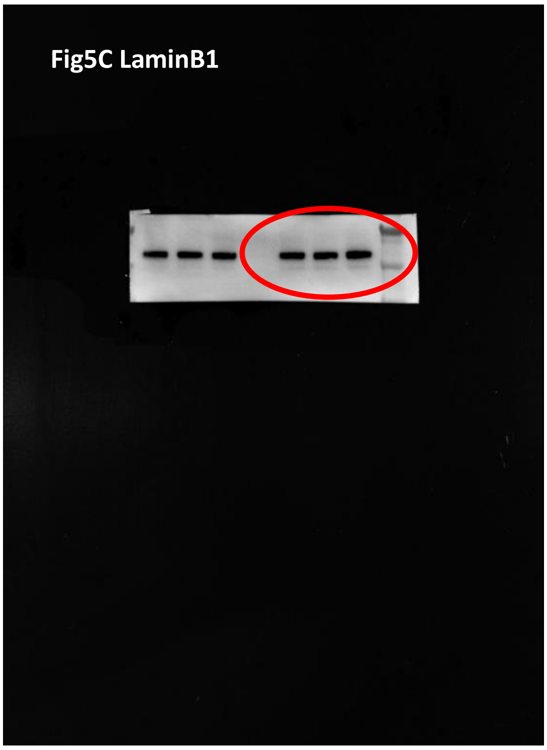
Fig5C LaminB1

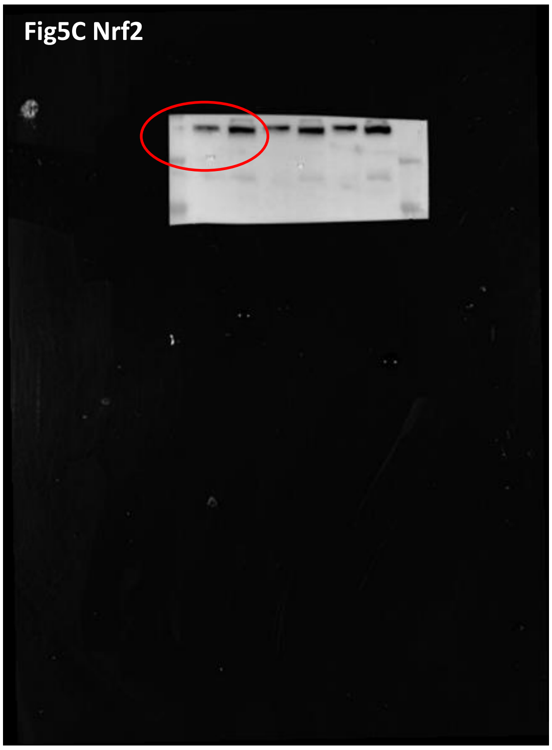
Fig5C Nrf2

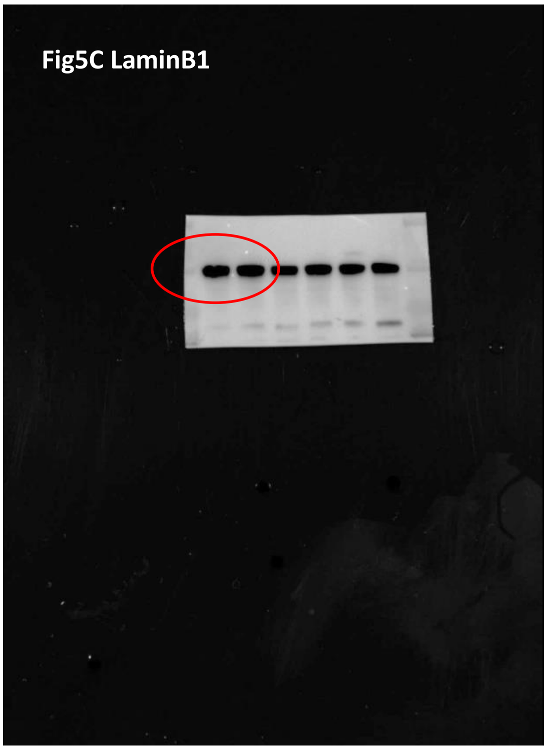
Fig5C LaminB1

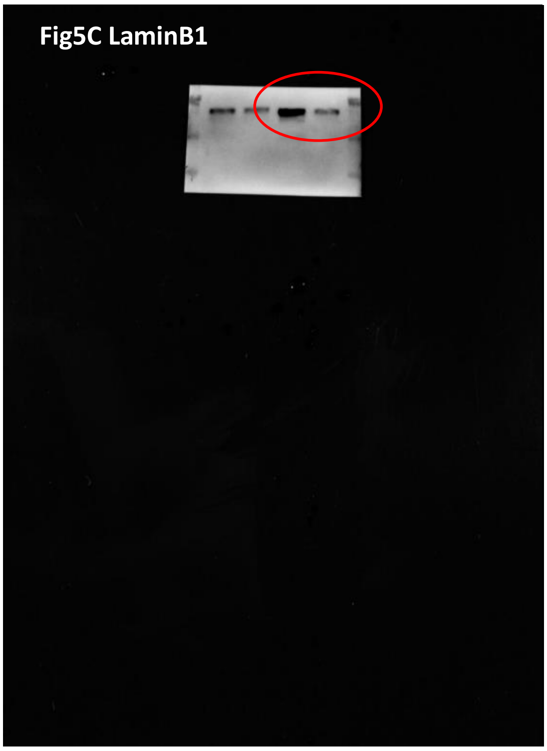
Fig5C LaminB1

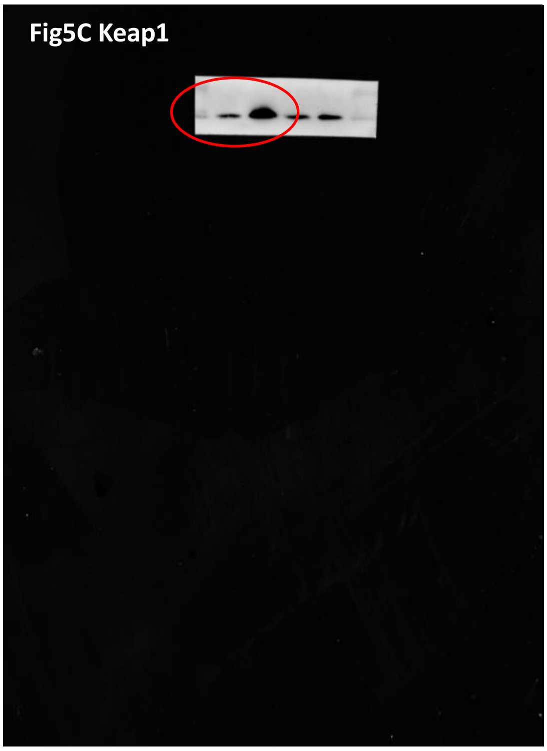
Fig5C Keap1

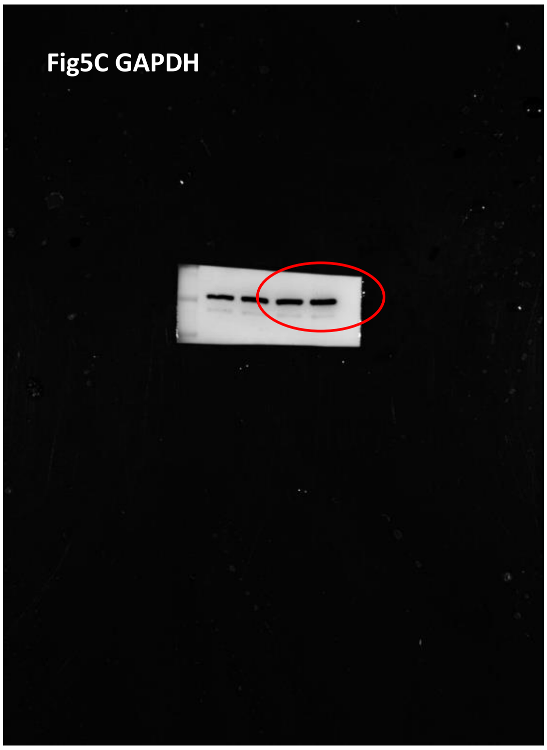
Fig5C GAPDH

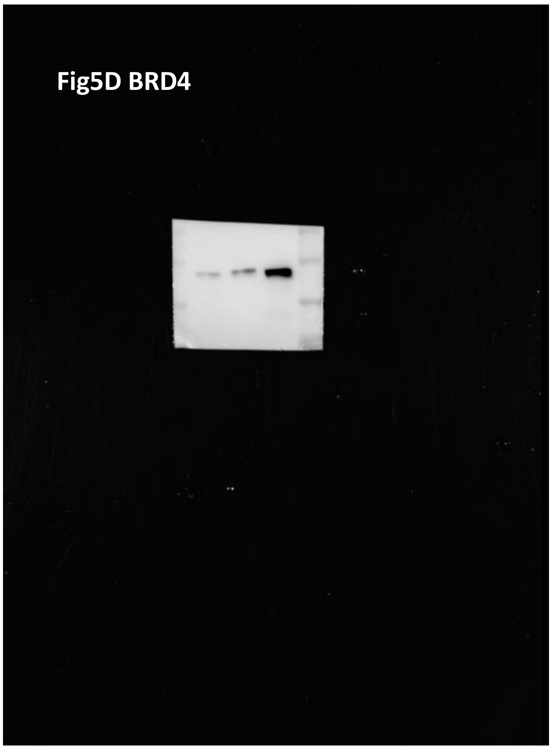
Fig5D BRD4

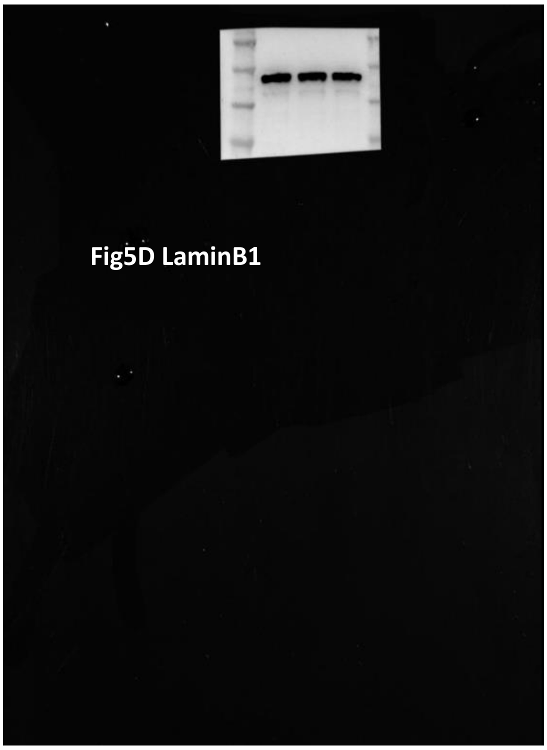
Fig5D LaminB1

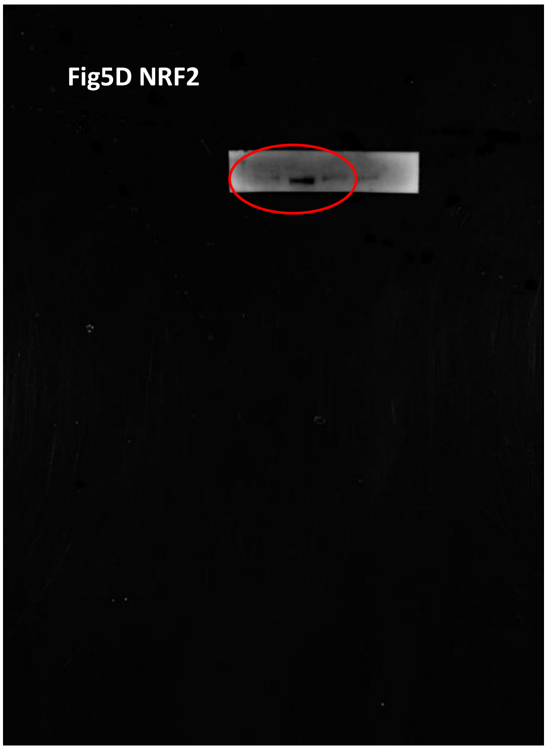
Fig5D NRF2

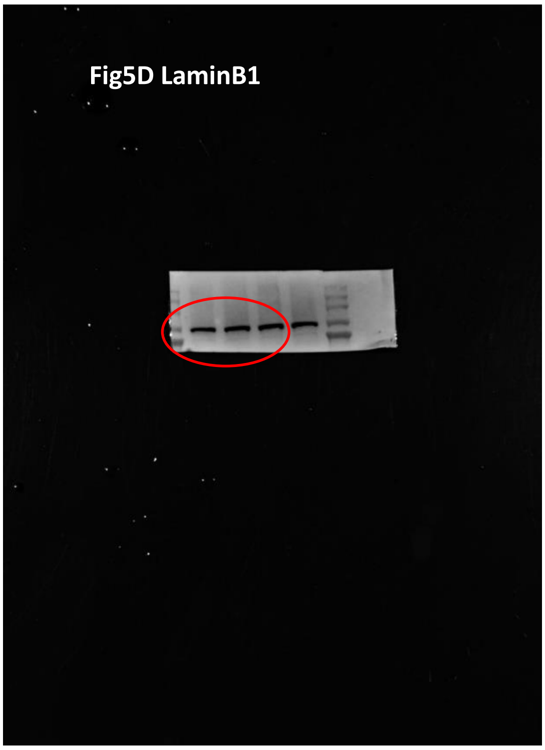
Fig5D LaminB1

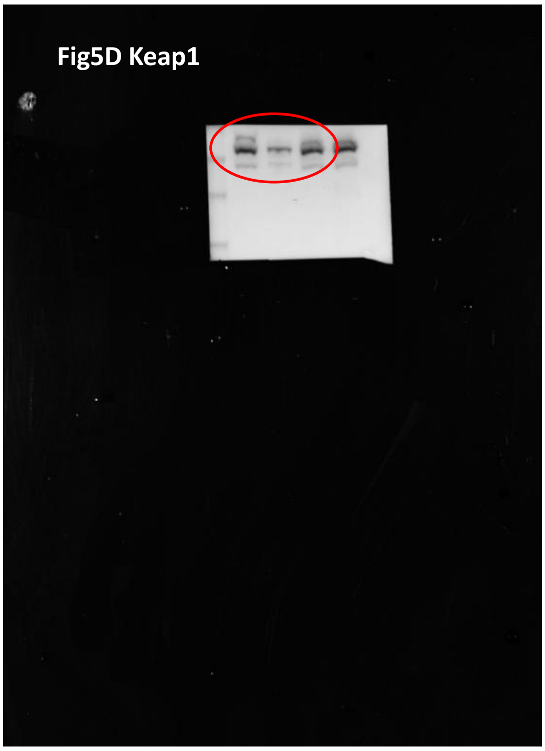
Fig5D Keap1

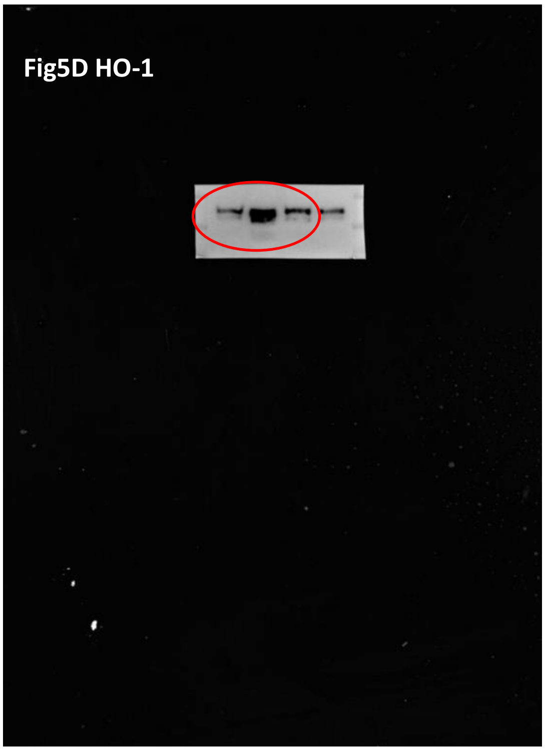
Fig5D HO-1

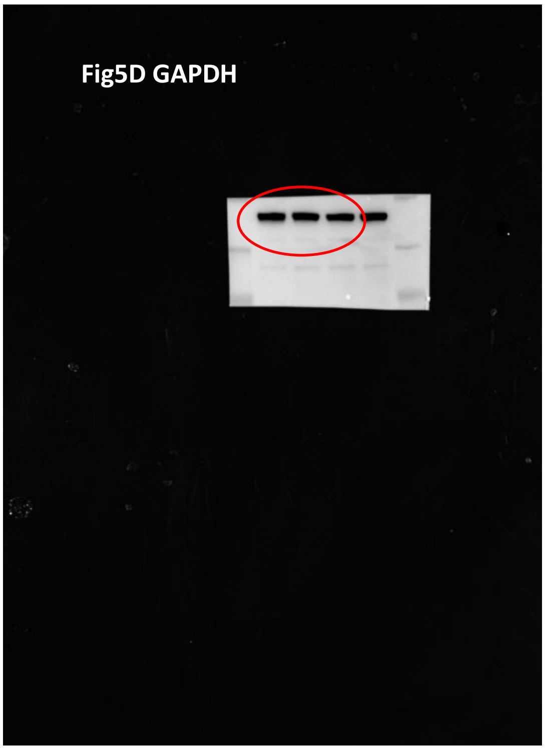
Fig5D GAPDH
